# Association between sleep disorders and subsequent heart failure

**DOI:** 10.1016/j.ijcha.2025.101618

**Published:** 2025-01-25

**Authors:** Jamschid Sedighi, Mark Luedde, Julia Gaensbacher-Kunzendorf, Hans-Joerg Hippe, Pascal Bauer, Birgit Assmus, Samuel Sossalla, Karel Kostev

**Affiliations:** a Medical Clinic I Cardiology and Angiology Justus-Liebig-University Giessen Germany; b Department of Cardiology Angiology and Intensive Care Medicine University Hospital of Schleswig Holstein Campus Kiel Germany; c Department of Cardiology Marien-Hospital Witten Witten Germany; d Christian-Albrechts-University of Kiel Germany; e Department of Cardiology Kerckhoff-Clinic Bad Nauheim Germany; f German Center for Cardiovascular Research (DZHK) Partner Site Rhine-Main Germany; g Epidemiology IQVIA Frankfurt Germany

**Keywords:** Sleep disorders, Heart failure, Coincidence

## Abstract

**Background:**

Sleep disorders are prevalent conditions that may influence the progression of various heart diseases. However, the relationship between sleep disorders and the onset of heart failure (HF) remains unclear.

**Objective:**

The purpose of this study was to investigate whether sleep disorders are associated with an increased risk of developing HF.

**Methods:**

We conducted a retrospective cohort study using data from 1,293 general practices across Germany sourced from the IQVIA Disease Analyzer database. The study included patients with an initial diagnosis of HF (ICD-10 code: I50) between January 2010 and December 2022 (index data) These patients were matched with control subjects without HF based on age, sex, and pre-existing conditions. The primary outcome was the association between prior sleep disorder diagnoses and the subsequent development of HF. Data were available for 9,345,246 individuals, of which 406,265 had a history of HF.

**Results:**

The study analyzed data from 123,516 patients with HF and an equal number of matched controls. The mean age of participants was 73.3–73.4 (SD 12.4) years, with 53.2% being women. Sleep disorders diagnosed prior to the onset of HF were significantly associated with an increased risk of developing HF. This association was consistent across different types of sleep disorders overall (OR: 1.22; 95%CI: 1.19–1.24) as well as insomnia (OR: 1.26; 95%CI: 1.21–1.31), sleep apnea (OR: 1.20; 95%CI: 1.15–1.25), and unspecified sleep disorders (OR: 1.21; 95%CI: 1.18–1.25).

**Conclusion:**

In this large cohort of outpatients, a prior diagnosis of sleep disorders was linked to a higher incidence of HF. These findings suggest that sleep disorders may serve as a risk factor for the development of HF, highlighting the need for early identification and management of sleep disturbances in at-risk populations. Addressing these disorders in clinical practice could represent a pivotal step towards better cardiovascular health and patient care.

## 1 Background

There is a growing awareness of the importance of sleep-related disorders in patients with cardiovascular diseases^1^. Patients with heart failure (HF) in particular often exhibit sleep-related breathing disorders such as obstructive and central sleep apnea syndrome and they may have short sleep duration and poor sleep quality [1]. These problems even appear to be relevant for the prognosis of those affected [2]. It is still unclear whether sleep disorders are also risk factors for the occurrence of HF.

We aimed to examine whether a history of sleep disorders was more frequent in patients with HF as compared to those without an HF diagnosis. We included both sleep apnea syndrome and sleep disorders such as insomnia.

## 2 Methods

This study is based on data from German primary care practices from the publicly available Disease Analyzer database (IQVIA) [3]. This database contains anonymized longitudinal data on diagnoses as well as medical and demographic data taken directly from the computer system of a representative sample of practices throughout Germany. Patient diagnoses are entered and updated on an ongoing basis during outpatient visits. No distinction is made between inpatient and outpatient diagnoses. Mortality data are not part of the data collection. The study population included all patients aged ≥ 18 years with an initial HF diagnosis (ICD-10 code: I50) between January 2010 and December 2022 (index date) who had at least one year of observation prior to the index date. Controls were individuals without HF who were matched (1:1) by age, sex, their pre-diagnostic observation time in years, and pre-defined diagnoses that may be associated with increased risk of HF (**Supplementary Fig. 1**). The primary outcome of the study was the association between sleep disorders documented prior to the index date and subsequent HF diagnosis. Sleep disorders (ICD-10: G47) included insomnia, hypersomnia, circadian rhythm sleep disorders, sleep apnea, narcolepsy and cataplexy, parasomnia, sleep related movement disorders, and unspecified sleep disorders.

Our study was structured as a case-control study. To examine whether a history of sleep disorders was more frequent in patients with HF as compared to those without HF diagnosis, we used logistic regression models and estimated odds ratios (ORs) with 95 % confidence intervals (95 %CI) for sleep disorders across cases and controls. Our research protocol was evaluated by the local ethics committee of Christian-Albrechts-University (CAU) Kiel, Germany; since we used only anonymized data that could not be traced back to individual persons, it was confirmed that it was not necessary to obtain informed consent from individual patients to participate in the study (file reference D413/21 of the CAU ethics committee). Further methodological details can be found in the Supplementary Methods section.

## 3 Results

### 3.1 Baseline characteristics

After 1:1 matching, 123,516 HF cases and 123,516 non-HF controls were available for the analyses (**Supplementary Fig. 1**). The mean age (with standard deviation, SD) at the index date was 73.3–73.4 (12.4) years, and 53.2 % of the patients were women. On average, both cases and controls had 8.5 (SD 6.1) years of pre-observation time prior to the index date. Case and controls had the same proportions of pre-defined diagnoses (Table 1).

**Table 1:** Characteristics of study patients after 1:1 matching.

**Variable**	**Heart failure (n = 123,516)**	**Non-heart failure (n = 123,516)**	**Standardized mean difference**
*Age (in years)*			
Mean (±SD)	73.4 (±12.4)	73.3 (12.4)	0.001
<60	18,984 (15.4)	18,973 (15.4)	
60–69	22,786 (18.4)	22,777 (18.4)	
70–79	38,221 (30.9)	38,236 (31.0)	
80–89	38,145 (30.9)	38,152 (30.9)	
90+	5,380 (4.4)	5,378 (4.3)	
Sex			
Female	65,659 (53.2)	66,674 (53.2)	0.003
Male	57,857 (46.8)	57,842 (46.8)	
Observation time prior to the index date (years), mean (±SD)	8.5 (±6.1)	8.5 (6.1)	0.000
*Conditions documented prior to index date*			
Diabetes mellitus	38,758 (31.4)	38,749 (31.4)	0.000
Obesity	15,731 (12.7)	15,735 (12.7)	0.000
Lipid metabolism disorders	49,972 (40.5)	49,965 (40.5)	−0.004
Hypertension	89,930 (72.8)	89,927 (72.8)	0.010
Myocardial infarction	6,893 (5.6)	6,879 (5.6)	−0.010
Chronic ischemic heart disease	28,735 (23.3)	28,727 (23.3)	−0.003
Atrial fibrillation and flutter	15,738 (12.7)	15,722 (12.7)	−0.018
Chronic bronchitis / COPD	15,557 (12.6)	15,568 (12.6)	−0.010
Chronic kidney disease	10,408 (8.4)	10,416 (8.4)	−0.011
Cancer	13,614 (11.0)	13,623 (11.0)	−0.002

Data are absolute sample numbers and percentages, n(%), unless otherwise specified.

SD = standard deviation, COPD = chronic obstructive pulmonary disease.

### 3.2 Association between sleep disorders and subsequent HF diagnosis

Sleep disorders documented any time prior to the index date were associated with an increased risk of subsequent HF. This association was observed for sleep disorders in total (OR: 1.22; 95 %CI: 1.19–1.24) as well as insomnia (OR: 1.26; 95 %CI: 1.21–1.31), sleep apnea (OR: 1.20; 95 %CI: 1.15–1.25) and undefined sleep disorder (OR: 1.21; 95 %CI: 1.18–1.25) (Table 2). There were no sex-specific differences in these associations. In age-stratified analyses, the association between sleep disorders overall and HF was significant in all age groups, with ORs between 1.18 and 1.26. Association with insomnia was only significant in patients aged 70 years and older, and with sleep apnea the association was significant in age groups < 60, 60–69, and 70–79 years but not in patients > 80 years (Table 2). Among patients diagnosed with HF, 71.5 % received at least one prescription of HF-related medication including diuretics, beta-blockers, ACE inhibitors, or angiotensin receptor blockers.

**Table 2:** Association between sleep disorders and subsequent HF diagnosis.

	Odds Ratios (95 % Confidence Intervals) for sleep disorder documented any time prior to index date compared to no sleep disorder							
Sleep disorder type	All patients	<60 years	60–69 years	70–79 years	80–89 years	90 + years	Women	Men
Sleep disorders total	1.22 (1.19.1.24)*	**1.18 (1.11**–1.25)*	**1.19 (1.13**–1.26)*	**1.21 (1.16**–**1.26)**	**1.25 (1.20**–1.30)*	**1.26 (1.12**–1.41)*	**1.22 (1.18**–1.25)*	**1.22 (1.18**–1.26)*
Insomnia	**1.26 (1.21**–1.31)*	1.14 (1.02–1.28)	1.16 (1.04–1.28)	**1.30 (1.21**–1.41)*	**1.29 (1.20**–1.39)*	**1.46 (1.21**–1.77)*	**1.26 (1.20**–1.33)*	**1.26 (1.17**–1.35)*
Sleep apnea	**1.20 (1.15**–1.25)*	**1.32 (1.20**–1.45)*	**1.23 (1.13**–1.32)*	**1.17 (1.09**–1.26)*	1.10 (1.01–1.21)	0.92 (0.57–1.47)	**1.19 (1.11**–1.28)*	**1.20 (1.14**–1.28)*
Sleep disorder undefined	**1.21 (1.18**–1.25)*	1.10 (1.02–1.19)	**1.19 (1.11**–1.28)*	**1.19 (1.13**–1.26)*	**1.28 (1.22**–1.36)*	1.21 (1.06–1.38)	**1.21 (1.17**–1.25)*	**1.22 (1.16**–1.28)*

Abbreviations: OR = odds ratio; CI = confidence interval.

*p < 0.001.

Most of the patients (>95 %) were on a combination therapy of at least two different drugs. Considering separate drug classes, 66.9 % of HF patents received diuretics, 45.7 % beta-blockers, 33.7 % ACE inhibitors, and 27.4 % angiotensin receptor blockers.

### 3.3 Dependence of association between sleep disorders and HF on the time of sleep disorder documentation

Table 3 shows results of further regression models investigating the impact of periods of sleep disorder diagnosis and HF. The strongest association with HF were observed for sleep disorders documented within one year prior to the index date (OR: 1.28; 95 %CI: 1.23–1.33 for sleep disorders overall; OR: 1.22; 95 %CI: 1.13–1.32 for insomnia, OR: 1.35; 95 %CI: 1.24–1.46 for sleep apnea). The strength of the associations declined with increased distance to the index date and became not significant either in the second or in the fourth year prior to the index date (Table 3).

**Table 3:** Association between time period of sleep disorders prior to index date and subsequent HF diagnosis.

	Odds Ratios (95 % Confidence Intervals) for sleep disorder documented in defined time compared to no sleep disorder							
Documentation time prior to index date	All patients	<60 years	60–69 years	70–79 years	80–89 years	90 + years	Women	Men
Sleep disorders total								
≤1 year	**1.28 (1.23**–1.33)*	**1.25 (1.13**–1.38)*	**1.31 (1.19**–1.43)*	**1.28 (1.19**–1.37)*	**1.27 (1.19**–1.37)*	**1.36 (1.12**–1.66)*	**1.25 (1.19**–1.32)*	**1.32 (1.24**–1.40)*
2–––3 years	**1.09 (1.05**–1.13)*	1.08 (0.98–1.18)	1.13 (1.04–1.23)	1.05 (0.98–1.12)	1.09 (1.02–1.17)	1.12 (0.94–1.34)	**1.09 (1.04**–1.15)*	1.08 (1.02–1.14)
4–––5 years	1.05 (1.00–1.09)	1.07 (0.96–1.20)	1.01 (0.92–1.12)	1.06 (0.98–1.14)	1.04 (0.96–1.13)	1.08 (0.86–1.35)	1.04 (0.98–1.10)	1.06 (0.99–1.13)
>5 years	1.05 (1.01–1.08)	1.12 (1.02–1.24)	1.00 (0.92–1.08)	1.04 (0.98–1.11)	1.03 (0.97–1.10)	1.17 (0.97–1.41)	1.05 (1.00–1.10)	1.04 (0.98–1.10)
Insomnia								
≤1 year	**1.22 (1.13**–1.32)*	1.09 (0.88–1.34)	1.16 (0.94–1.43)	**1.35 (1.18**–1.56)*	1.19 (1.05–1.36)	1.16 (0.82–1.64)	**1.19 (1.08**–1.31)*	**1.28 (1.12**–1.46)*
2–––3 years	**1.17 (1.09**–1.26)*	1.22 (1.00–1.49)	1.33 (1.11–1.59)	1.13 (0.99–1.29)	1.11 (0.98–1.25)	1.19 (0.86–1.63)	1.13 (1.03–1.23)	**1.25 (1.11**–1.42)*
4–––5 years	1.02 (0.93–1.11)	1.07 (0.83–1.37)	0.93 (0.74–1.16)	1.04 (0.89–1.21)	1.02 (0.88–1.19)	1.12 (0.74–1.69)	1.03 (0.92–1.14)	1.02 (0.87–1.18)
>5 years	1.11 (1.04–1.19)	1.13 (0.93–1.39)	1.01 (0.86–1.18)	1.12 (0.99–1.26)	1.11 (0.98–1.25)	1.70 (1.22–2.36)	1.16 (1.06–1.26)	1.04 (0.92–1.17)
Sleep apnea								
≤1 year	**1.35 (1.24**–1.46)*	**1.54 (1.30**–1.83)*	**1.33 (1.14**–1.54)*	**1.40 (1.21**–1.62)*	1.11 (0.91–1.36)	0.38 (0.11–1.30)	**1.29 (1.11**–1.49)*	**1.38 (1.25**–1.52)*
2–––3 years	1.06 (0.99–1.14)	1.06 (0.91–1.23)	1.05 (0.93–1.20)	1.05 (0.93–1.18)	1.09 (0.92–1.28)	1.59 (0.64–3.97)	1.10 (0.97–1.23)	1.05 (0.96–1.14)
4–––5 years	1.08 (1.00–1.17)	1.05 (0.87–1.27)	1.17 (1.00–1.37)	1.02 (0.88–1.17)	1.10 (0.90–1.33)	1.30 (0.44–3.80)	1.05 (0.90–1.23)	1.09 (0.99–1.21)
>5 years	1.06 (0.99–1.14)	1.22 (1.02–1.46)	1.03 (0.91–1.18)	1.06 (0.94–1.19)	1.02 (0.87–1.18)	0.98 (0.48–1.98)	1.09 (0.96–1.24)	1.05 (0.97–1.14)
Sleep disorder undefined								
≤1 year	**1.26 (1.20**–1.33)*	1.11 (0.88–1.15)	**1.32 (1.15**–1.50)*	**1.18 (1.07**–1.30)*	**1.34 (1.22**–1.17)*	**1.55 (1.23**–1.96)*	**1.26 (1.28**–1.35)*	**1.27 (1.17**–1.39)*
2–––3 years	1.06 (1.01–1.12)	1.00 (0.88–1.15)	1.14 (1.01–1.28)	1.04 (0.95–1.13)	1.07 (0.98–1.17)	1.03 (0.83–1.27)	1.07 (1.00–1.14)	1.05 (0.97–1.14)
4–––5 years	1.06 (1.00–1.12)	1.19 (1.01–1.40)	0.95 (0.82–1.09)	1.10 (0.99–1.22)	1.03 (0.93–1.14)	1.13 (0.86–1.48)	1.06 (0.98–1.13)	1.07 (0.97–1.19)
>5 years	1.05 (1.00–1.10)	1.10 (0.96–1.26)	1.00 (0.90–1.12)	1.05 (0.96–1.14)	1.06 (0.98–1.15)	1.04 (0.83–1.31)	1.04 (0.99–1.11)	1.06 (0.98–1.15)

Abbreviations: OR = odds ratio; CI = confidence interval.

*p < 0.001.

## 4 Discussion

The present study investigated the association between sleep disorders and new-onset of HF in a large cohort of outpatients in Germany. Sleep disorders were significantly associated with an increased incidence of HF but only when sleep disorders occurred within one year prior to HF diagnosis.

Interestingly, the statistical association between sleep disorders and HF is particularly strong within one year prior to diagnosis. This could be an indication that there is a reciprocal influence between the two entities: on the one hand, a possible induction of HF by sleep disorders, but on the other hand, the first symptoms may also show up first in the year before the diagnosis of HF, such as nocturnal coughing, dyspnea when lying supine, etc. Both effects may partly explain the statistical relationship. In the year before initial diagnosis, the aspect of sleep disorders as a possible early symptom of HF may even predominate.

The current findings are consistent with the limited prior research investigating the relationship between sleep disorders and HF. For example, Gottlieb et al. conducted a prospective study as part of the Sleep Heart Health Study, which demonstrated a strong association between obstructive sleep apnea and the risk of incident HF [4]. Similarly, Javaheri et al. provided updated insights into the interactions between obstructive sleep apnea and cardiovascular disease pathophysiology, further corroborating the results of this study [5].

Additionally, Piccirillo et al. and Spiesshoefer et al. highlighted the underappreciated role of sleep disturbances in cardiovascular diseases, emphasizing both their contribution to disease progression and their potential as therapeutic targets [6], [7]. While these studies focus broadly on cardiovascular outcomes, the present study narrows its focus to HF, providing more specific evidence for the association with sleep apnea and insomnia (Table 4).

**Table 4:** Comparison of Relevant Studies.

**Study**	**Year**	**Design**	**Population Size**	**Exposure Variable**	**Outcome Variable**	**Key Findings**
Gottlieb et al.	2010	Prospective	5,423	Sleep apnea	HF, coronary heart disease	Sleep apnea significantly increased HF risk (HR: 1.58)
Javaheri et al.	2024	Review	N/A	Sleep apnea	Cardiovascular disease	Emphasized mechanistic links between sleep apnea and HF
Piccirillo et al.	2023	State-of-the-Art	N/A	Sleep apnea	HF	Highlighted sleep apnea as a critical HF risk factor
Spiesshoefer et al.	2021	Clinical Review	N/A	Sleep disturbances	Cardiovascular disease	Discussed sleep as an underestimated factor in cardiovascular health
Present Study	2025	Retrospective	123,516 pairs	Sleep apnea, insomnia	HF	Sleep apnea (OR: 1.20) and insomnia (OR: 1.26) linked to HF

This study also considers the possibility of reverse causality, wherein HF contributes to the onset of sleep disorders rather than vice versa. Mansfield et al. demonstrated that successful HF treatment via heart transplantation reduced central sleep apnea, supporting this bidirectional relationship. Similarly, Arzt et al. identified a high prevalence of sleep disturbances among patients with systolic HF, suggesting that HF symptoms may exacerbate or induce sleep disorders such as sleep apnea and insomnia.

These studies collectively underscore the bidirectional relationship between HF and sleep disorders. Addressing sleep disorders early, particularly before HF becomes symptomatic, could not only improve sleep health but potentially mitigate the progression of HF.

It should be borne in mind that our study does not show causal relationships but rather statistical associations. Nevertheless, causal relationships that link sleep disorders to an increased risk to develop HF are conceivable: Some risk factors may apply to the two types of sleep-related breathing disorders (obstructive sleep apnea and central sleep apnea) and other sleep disorders such as insomnia and sleep disruption. These may include, for example, increased sympathetic innervation [8], [9], hypertension [10], and an elevated level of inflammatory cytokines [11], [12]. In addition to sympathetic innervation, multiple neurotransmitters are thought to play a role in sleep. These include serotonin from the dorsal raphe nucleus, norepinephrine contained in neurons with cell bodies in the locus ceruleus, and acetylcholine from the pontine reticular formation [13]. Dysregulation of these systems has thus far mainly been associated with the development of neurological diseases [14]. This could also provide an interesting field for research into the connection with heart disease.

Our study highlights the critical need for identifying and screening for sleep disorders clinically in those at higher risk of HF. Automated sleep assessments using digital devices could lead to better outcomes for HF patients when incorporated into routine care. Risk stratification based on sleep disorders could be improved, and patients should be educated about the association between HF and sleep health.

### 4.1 Limitations

Some limitations of our study need to be considered. Data are based on ICD-10 codes only. Information is lacking about the severity of disease and the respective treatment status of the patients, both with regard to the various sleep disorders and HF. In the control group, data from a sleep laboratory are not available for all patients, so that we cannot rule out the possibility that there are also patients with unrecognized sleep apnea syndrome. Based on the ICD-10 code, we cannot differentiate between the individual forms of HF (HF with reduced, moderately reduced, and preserved ejection fraction [15]). Sleep disorders are in most cases 'undefined' (G47.9), and only insomnia and sleep apnea could be identified separately. Different forms of sleep disorderes such as interrupted sleep, sleepwalking, nightmares or difficulty falling asleep could not be differentiated, which limits the ability to draw precise conclusions about their specific roles in the development of heart failure. This lack of granularity impacts the interpretability of the results and suggests a need for more detailed data collection in future studies.

Since our database does not offer data on the severity of cardiovascular disease, we cannot ensure that both groups are balanced in this regard. Furthermore, a lack of data on BNP/NT-proBNP levels precludes any meaningful assessment of the severity of HF.

The absence of psychological factors, such as anxiety and depression, in our study cohort limits the possibility that these two factors are associated with both HF and sleep disorders. Although we have chosen the model of a case-control study that is stratified with regard to as many influencing factors as possible, a confirmation bias, i.e. that we find an association because we are looking for it, cannot be completely ruled out*.* The fact that the association between sleep disorders and HF is particularly strong in the year before diagnosis of HF could also be related to increased ‘vigilance’ in the year of diagnosis. We tried to minimize this bias by matching cases and controls even after the pre-observation period. Nevertheless, a bias is ultimately possible.

## 5 Conclusions

In spite of its limitations, our study shows an important link between sleep disorders − a problem that is still underestimated in its frequency and medical importance − and HF, one of the most important cardiological diseases. Sleep disorders should be viewed not only as factors influencing the course of the disease but also as risk factors for the occurrence of HF. This could provide a new option for the primary prevention of HF.

## CRediT authorship contribution statement

**Jamschid Sedighi:** Writing – review & editing, Writing – original draft, Visualization, Validation, Supervision, Software, Resources, Project administration, Investigation, Funding acquisition, Formal analysis, Data curation, Conceptualization. **Mark Luedde:** Supervision, Funding acquisition, Formal analysis, Data curation, Conceptualization. **Julia Gaensbacher-Kunzendorf:** Validation, Methodology, Data curation. **Hans-Joerg Hippe:** Supervision, Investigation, Conceptualization. **Pascal Bauer:** Supervision, Data curation, Conceptualization. **Birgit Assmus:** Supervision, Data curation. **Samuel Sossalla:** Visualization, Validation, Supervision, Conceptualization. **Karel Kostev:** Writing – original draft, Visualization, Validation, Supervision, Software, Resources, Project administration, Methodology, Investigation, Funding acquisition, Formal analysis, Data curation, Conceptualization.

## Declaration of competing interest

The authors declare that they have no known competing financial interests or personal relationships that could have appeared to influence the work reported in this paper.
