# Supplementary material for: Association between sleep disorders and subsequent heart failure
Source: Int J Cardiol Heart Vasc. 2025 Jan 25;57:101618. doi: 10.1016/j.ijcha.2025.101618 (PMC11803254; doi:10.1016/j.ijcha.2025.101618)
Supplement: Supplementary Data 1 [file mmc1.docx]

**Supplementary Methods**

*Database*

This study used data from German primary care practices from the Disease Analyzer database (IQVIA). Details of the methodology have been published previously[^1^](#_ENREF_1). In brief, the Disease Analyzer database contains data on demographic variables, diagnoses and prescriptions obtained in general and specialized practices in Germany. Practices included in the database are selected according to the yearly statistics of the German Medical Association, which include information on physician’s age, specialty group, community size category, and German federal state. The database covers approximately 1300 customary practices in Germany. It has previously been shown that the panel of practices included in the Disease Analyzer database is representative of general and specialized practices in Germany[^1^](#_ENREF_1).

*Study population*

The study population included patients aged ≥18 years with an initial HF diagnosis (ICD-10 code: I50) between January 2010 and December 2022 (index date) who had at least one year of observation prior to the index date. Controls were individuals without HF who were matched (1:1) by age, sex, their pre-diagnostic observation time in years, and pre-defined diagnoses which may be associated with increased risk of HF. These diagnoses documented prior to the index date included diabetes mellitus (ICD-10: E10-E14), obesity (ICD-10: E66), lipid metabolism disorders (ICD-10: E78), hypertension (ICD-10: I10-I15), myocardial infarction (ICD-10: I21-I23), chronic ischemic heart disease (ICD-10: I25), atrial fibrillation and flutter (ICD-10: I48), chronic kidney disease (ICD-10: N18, N19), cancer (ICD-10: C00-C97), and chronic bronchitis and COPD (as possible proxy for smoking, ICD-10: J42-J44). For individuals without HF, the index date was a randomly selected visit date between January 2010 and December 2022. The flow diagram of study participants is shown in supplementary Figure

*Study outcome*

Outcome of the study was the association between sleep disorders documented prior to the index date and subsequent HF diagnosis. Sleep disorders (ICD-10: G47) included insomnia, hypersomnia, circadian rhythm sleep disorders, sleep apnea, narcolepsy and cataplexy, parasomnia, sleep related movement disorders, and unspecified sleep disorders. However, GPs documented unspecified sleep disorders (ICD-10: G47.9) in most cases, following by insomnia (ICD-10: G47.0), and sleep apnea (ICD-10: G47.3). Other ICD-10 codes were rarely documented. Based on the frequency of sleep disorder detail documentation, analyses were conducted for sleep disorders in total, insomnia, sleep apnea, and unspecified sleep disorders.

*Statistical analyses*

Demographic and clinical characteristics of cases and controls after 1:1 propensity-score matching was evaluated using the t- test for continuous variables, the McNemar test for categorical variables with two categories, and the Stuart-Maxwell test for categorical variables with more than two categories. To examine whether a history of sleep disorders was more frequent in patients with HF, as compared to those with HF diagnosis, we used logistic regression models and estimated odds ratios (ORs) with 95% confidence intervals (95%CI) for sleep disorders across cases and controls. First model was conducted for the association between sleep disorder diagnosis any time prior to index date and subsequent HF diagnosis. In the second model, sleep disorders were classified based on the time of documentation: within one year, two to three, four to five, and more than five years prior to index date. Models were additionally calculated separately for five age groups, as well as by sex. To account for multiple comparisons and due to large patient samples, p-value <0.001 was considered statistically significant. All analyses were conducted using SAS version 9.4 (SAS institute, Cary, US).

**References**

1. Rathmann W, Bongaerts B, Carius HJ, Kruppert S, Kostev K. Basic characteristics and representativeness of the German Disease Analyzer database. *Int J Clin Pharmacol Ther.* 2018;56(10):459-466.
